# Real-world insights into neurodevelopmental outcomes amongst people with congenital hyperinsulinism

**DOI:** 10.1186/s13023-026-04471-7

**Published:** 2026-07-03

**Authors:** Lauren N. Lopez, Indraneel Banerjee, Diva D. De Leon, Tai L. S. Pasquini, Marcia Roeper, Kristen E. Rohli, Elizabeth Rosenfeld, Paul Thornton, Julie Raskin

**Affiliations:** 1 Congenital Hyperinsulinism International, Glen Ridge, NJ USA; 2 Department of Paediatric Endocrinology, Royal Manchester Children’s Hospital, Manchester, UK; 3 Congenital Hyperinsulinism Center, Division of Endocrinology and Diabetes, Children’s Hospital of Philadelphia, Philadelphia, PA USA; 4 Department of Pediatrics, Perelman School of Medicine, University of Pennsylvania, Philadelphia, PA USA; 5 Department of General Pediatrics, Neonatology and Pediatric Cardiology, Medical Faculty, University Hospital Düsseldorf, Heinrich-Heine-University, Düsseldorf, Germany; 6 Congenital Hyperinsulinism Center, Division of Endocrinology, Cook Children’s Medical Center, Fort Worth, TX USA

**Keywords:** Hyperinsulinism, Hypoglycemia, Neurodevelopmental challenges, Neurodevelopmental outcomes, Developmental delay, Registry, Natural history study

## Abstract

**Background:**

Congenital hyperinsulinism (HI) is a rare condition causing excessive insulin secretion, leading to severe hypoglycemia and high risk of neurological damage. Studies of neurodevelopmental outcomes in HI report prevalence ranging from less than one-quarter to about half of all people with HI. These studies largely focus on formal clinical assessments, but the real-world experiences of individuals with HI and their families are rarely published. The aim of this study was to describe the neurodevelopmental outcomes of a heterogenous cohort of individuals with HI directly from the perspective of the person with HI or their family, as shared through the HI Global Registry.

**Results:**

193 participants with HI from 34 countries were included in this analysis. Mean age at follow-up was 12.52 years (SD = 12.60). 66% of all participants, and 72% of participants aged ≥ 5 years at follow-up (*n* = 145), reported an adverse neurodevelopmental outcome. Of those aged ≥ 5 years at follow-up, 50% reported a diagnosed neurological, neurodevelopmental, or sensory disorder, 57% reported a history of developmental delay, and 18% reported a diagnosed mental health condition. Amongst participants ≥ 5 years of age with an adverse neurodevelopmental outcome, approximately half were capable of performing at peer level in domains of daily life. Participants added richness to the categorical data by providing free-text responses describing the problems faced in day-to-day life, with reported challenges in social, emotional, and behavioral issues.

**Conclusions:**

This is the first study of neurodevelopmental outcomes in people with HI to focus on real-world data reported directly from people with HI and their families. Neurodevelopmental challenges were highly prevalent in people with HI and extended beyond formal clinical diagnoses into social, emotional, and behavioral problems. Proactive developmental assessments should be offered to all children with HI, and efforts should be made to develop multidisciplinary teams to support all medical and developmental aspects of care for people with HI. The real-world data on neurodevelopmental outcomes and experiences of individuals with HI and their families generated in this study will serve as a benchmark and help to quantify how future advances in diagnosis and treatment impact patient-centered neurodevelopmental outcomes.

**Supplementary Information:**

The online version contains supplementary material available at https://doi.org/10.1186/s13023-026-04471-7.

## Background

Congenital hyperinsulinism (HI) is a rare condition characterized by excessive insulin secretion due to dysfunctional pancreatic beta cells. It is the most common cause of persistent hypoglycemia in newborns, with an estimated prevalence of 1: 28,000 in European-ancestry populations [1]. Perinatal stress HI, sometimes referred to as transient HI, is typically caused by perinatal stress risk factors such as prematurity, intrauterine growth restriction, emergent delivery, etc. [2]., and typically resolves within 6 months of life, whereas persistent HI persists into childhood and sometimes adulthood. Persistent HI is caused by variants in at least 30 known genes [3], however around half of individuals with a clinical diagnosis of HI do not have an identified genetic cause [3–5].

The normal physiological response to hypoglycemia involves a cascade of responses to increase the metabolic fuel supply to the brain. However, in hyperinsulinism, failure to suppress insulin secretion in response to hypoglycemia inhibits the normal fasting mechanisms, including glycogenolysis, gluconeogenesis and the generation of ketones, an alternative brain fuel [6]. Regardless of the type of HI, hypoketotic hypoglycemia confers an imminent risk of permanent neurological damage [7, 8].

The neurological consequences of hyperinsulinism, including cerebral palsy, hearing and vision impairment, developmental delays, behavioral problems, etc., have long been recognized [9, 10]. However, the impact of the timing, duration, and severity of hypoglycemia on the risk of neurological damage is still not fully understood.

In recent years, research and advocacy efforts have focused on improving outcomes through the development of awareness campaigns and national and international guidelines for the diagnosis and management of neonatal hypoglycemia and persistent hyperinsulinism [11–15]. However, studies continue to report a high prevalence of adverse neurodevelopmental outcomes amongst people with HI. There is no standard definition of an adverse neurodevelopmental outcome in HI, and published studies all report on different diagnoses and evaluations. Different neurological and developmental evaluation tools are used across studies, making comparisons between studies challenging. Many studies report rates of epilepsy, but not all studies report other diagnoses, such as cerebral palsy, hearing impairment, vision impairment, autism spectrum disorder, learning disability, etc., leading to a potential underestimation of the true extent of adverse neurological challenges amongst people with HI. As such, the published literature is highly variable, with reports on the prevalence of adverse neurodevelopmental outcomes ranging from less than 25% [16–18] up to 53% [2, 19–25]. Most of the previous studies on neurodevelopmental outcomes in HI provide observational information of a cross-sectional nature. There is currently no cohort-based longitudinal outcome study.

The majority of previous studies reporting neurodevelopmental outcomes in people with HI largely focus on formal clinical assessments and many report data from only a single medical center, however these studies may underestimate the extent of neurodevelopmental impairment and the impact on daily life for families. Studies that focus purely on clinical assessments may not capture the breadth of challenges experienced by people with HI and their families, such as symptoms of undiagnosed medical conditions, mental health disorders, challenges with behavior, and difficulties in school and with social interactions. There is therefore an urgent need for additional real-world and patient-centric data to complement the clinical data. The value of patient- and caregiver-reported data is increasingly being recognized [26, 27] and should be incorporated into the body of literature reporting on patient-centered outcomes; especially in rare diseases where outcomes may be less well clinically defined.

The current study reports on the neurodevelopmental outcomes of a large multi-national cohort of individuals with HI, as reported by participants with HI or their caregivers. The aim of this study was to describe the neurodevelopmental outcomes of a heterogenous cohort of individuals who were diagnosed with HI directly from the perspective of the person with HI or their family. This report incorporates free text quotes from respondents, adding context and nuance to the impact of the neurodevelopmental consequences of hypoglycemia on the life of individuals living with HI.

## Methods

**Fig. 1:**
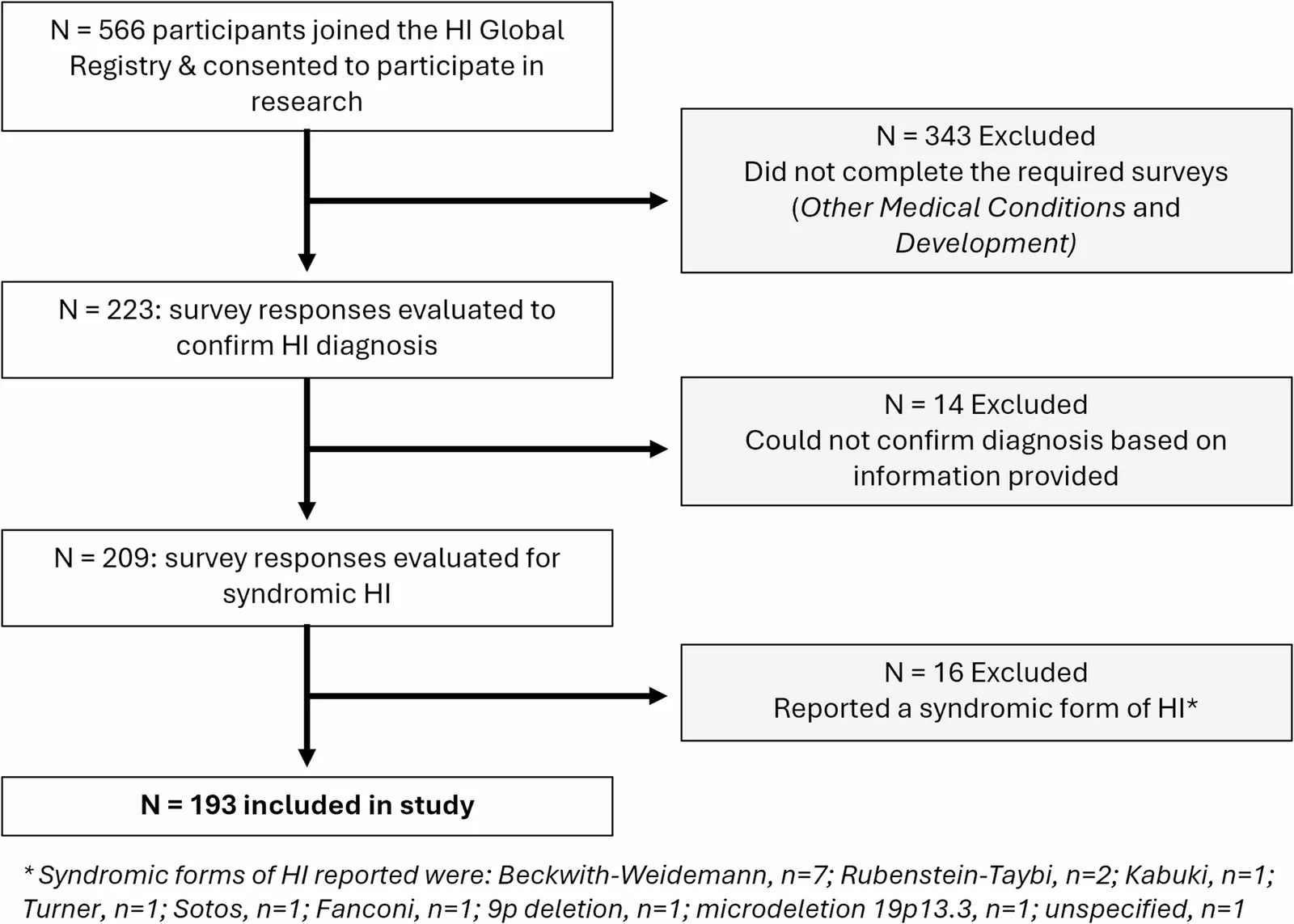
Inclusion-exclusion flow chart of study participants

Individuals who participated in the HI Global Registry (HIGR) [28, 29] were eligible to be included in this study. Data was collected from the time the registry launched in October 2018 to July 2025. Individuals who were included in this analysis consented to participate in research through HIGR, completed the two required surveys for this study (*Other Medical Conditions* and *Development*), and had a confirmed diagnosis of HI. A confirmed diagnosis of HI was defined as having at least one of the following: (1) A physician completed a survey sharing HI-related information from the participant’s medical record; (2) The participant reported taking at least one medication for HI (diazoxide or a somatostatin analog); (3) The participant had received a pancreatectomy for HI; and/or (4) The participant had received genetic testing for HI (results could be positive or negative). These criteria helped to exclude any participants who joined the registry because they suspected they had HI but were never treated or managed clinically for HI (Fig. 1). Individuals who reported having a syndromic form of HI were excluded.

### Neurodevelopmental outcomes

Participants with HI were assigned to the following neurodevelopmental outcome groups based on information shared in the *Other Medical Conditions* and *Development* surveys:

#### Adverse neurodevelopmental outcome

If the respondent reported any of the following (regardless of current age):

- A **diagnosis** of cerebral palsy, epilepsy, hearing impairment/deafness, vision impairment/blindness, intellectual disability, attention deficit hyperactivity disorder (ADHD), autism spectrum disorder (ASD), developmental coordination disorder, language disorder / auditory processing disorder, learning disability, or movement or tic disorder.
- **Delays in developmental milestones** (not necessarily clinically diagnosed) including social/emotional milestones, language/speech/communication milestones, cognitive milestones, gross motor milestones, fine motor milestones, or coordination.
- The following **other neurodevelopmental problems** (not necessarily clinically diagnosed conditions): hypotonia, movement disorder, learning disability, poor coordination, behavior problems, sensory processing problems, memory processing problems.

#### Normal neurodevelopment

If none of the above-listed conditions were reported and the participant was at least 5 years of age at follow-up.

#### Too young to assess

If none of the above listed conditions were reported but the participant was under 5 years of age at follow-up. The age of 5 years was selected because most children start school at this age, and this is when a child’s development compared to their peers may become more noticeable to caregivers. Prior to this age, more subtle neurodevelopmental differences may not be as obvious.

### Age at follow-up

The age at follow-up was defined as the age at submission of the most recent check-in survey, or the age at submission of the most recent *Other Medical Conditions* survey, which collects data on diagnosed conditions.

### Data analysis

The most recent survey submission was used for data analysis if participants had submitted a survey multiple times, i.e., in the case of survey updates or longitudinal survey retakes. Categorical data was collected via multiple-choice questions with pre-defined answer options. Categorical data were reported using summary statistics. Data analysis was conducted using STATA v18.5.

A free text box was provided for “other” responses that did not fit into pre-defined options or for the respondent to elaborate on additional details related to the survey topic. Free text responses that were written in a non-English language were translated using DeepL online software [30]. Free text responses were coded into themed categories where appropriate, and results were summarized as percentage of comments that mentioned a given theme. Free text quotes described in this manuscript were edited for spelling, grammar, and clarity where necessary.

### Further information about the HI Global Registry

HIGR is an international patient registry for people with HI, launched in 2018. People with HI (age 18 + years) or the parents or caregivers of people with HI (age < 18 years or 18 + years and unable to consent to research) share details about their history and experiences with HI via registry surveys. Regardless of who entered the data (person with HI or parent/caregiver), all data presented in this study refers to the person with HI. Each survey focuses on a different aspect of life with HI and surveys have been described in more detail elsewhere [28, 29]. The registry is available in eight languages and has enrolled participants from over 60 countries to date [28]. Participants are recruited on an ongoing basis via multiple channels, including emails, social media, conferences, and physician referrals. As of April 2025, participants who complete all required surveys receive a $50 gift card to thank them for their time and effort spent contributing to HI research.

HIGR is a research study that was approved under the North Star Institutional Review Board (NB100012) to ensure that all study activities comply with legal and ethical standards.

## Results

### Study participants

A total of 193 individuals with HI were included in the analysis (Fig. 1). Participants represented an international cohort from 34 countries (55% United States) (Table 1). 80% of participants had data reported on their behalf by a caregiver, of which 95% were children < 18 years and 5% were adults who did not have the capacity to consent to research. 88% of participants with a non-KATP channel mutation had an adverse neurodevelopmental outcome compared to 61% of those with a KATP mutation. Non-KATP mutations reported included *GLUD1* (*n* = 12 adverse outcome, *n* = 1 normal outcome ≥ 5 years), *GCK* (*n* = 3 adverse outcome, *n* = 1 normal outcome ≥ 5 years), *HNF4A* (*n* = 3 adverse outcome, *n* = 1 normal outcome < 5 years), *HNF1A* (*n* = 2 adverse outcome), and *UCP2*^1^ (*n* = 1 adverse outcome).

**Table 1:** Basic demographics of study participants

	Neurodevelopmental outcome			
	**Adverse** (*n* = 127)	**Normal at age ≥ 5 years** (*n* = 40)	**< 5 years (Excluded)** (*n* = 26)	**Total** (*n* = 193)
**Sex**				
Female	70 (69%)	22 (22%)	10 (10%)	102 (100%)
Male	57 (63%)	18 (20%)	16 (18%)	91 (100%)
**Age at Follow-up**				
Mean, years (SD)	12.75 (11.96)	18.68 (14.27)	1.92 (1.44)	12.52 (12.60)
**Surveys completed By**				
Participant with HI (adult)	25 (66%)	13 (34%)	0 (0%)	38 (100%)
Caregiver on behalf of participant with HI	102 (66%)	27 (17%)	26 (17%)	155 (100%)
**HI type**				
Diffuse	71 (68%)	24 (23%)	10 (10%)	105 (100%)
Focal	6 (43%)	5 (36%)	3 (21%)	14 (100%)
Atypical	4 (67%)	2 (33%)	0 (0%)	6 (100%)
Other	11 (85%)	2 (15%)	0 (0%)	13 (100%)
Unknown / Not reported	35 (64%)	7 (13%)	13 (24%)	55 (100%)
**HI genetics**				
KATP mutation	39 (61%)	14 (22%)	11 (17%)	64 (100%)
Non-KATP mutation	21 (88%)	2 (8%)	1 (4%)	24 (100%)
No identified genetic mutation	28 (61%)	8 (17%)	10 (22%)	46 (100%)
Not tested / Not reported	39 (66%)	16 (27%)	4 (7%)	59 (100%)
**Pancreatectomy status**				
Pancreatectomy ≥ 75%	18 (67%)	9 (33%)	0 (0%)	27 (100%)
Pancreatectomy < 75%	9 (56%)	5 (31%)	2 (13%)	16 (100%)
No pancreatectomy	97 (66%)	25 (17%)	24 (16%)	146 (100%)
Unknown / Not reported	3 (75%)	1 (25%)	0 (0%)	4 (100%)

### Neurodevelopmental outcomes

66% of individuals with HI (of which 83% ≥ 5 years old) reported an adverse neurodevelopmental outcome (Table 2). Of all study participants who were ≥ 5 years old at the time of follow up (*n* = 145), 105 (72%) reported an adverse neurodevelopmental outcome. One-fifth of participants were ≥ 5 years old at the time of follow-up and reported normal neurodevelopment, and 13% of participants were < 5 years and reported normal neurodevelopment.

**Table 2:** Composite neurodevelopmental outcome

	Age at Follow-up		
	**< 5 years** (*n* = 48)	**≥ 5 years** (*n* = 145)	**Total** (*n* = 193)
Adverse neurodevelopmental outcome	22 (11%)	105 (54%)	127 (66%)
Normal neurodevelopmental outcome	26 (13%)	40 (21%)	66 (34%)

40% of participants reported a diagnosed neurological, neurodevelopmental, or sensory disorder (Table 3). The most frequent reported diagnoses were epilepsy (16%), ADHD (16%), and learning disability (14%). Of those who reported epilepsy, all reported taking seizure medication previously (*n* = 1 < 5 years at follow-up; *n* = 3 ≥ 5 years at follow-up) or currently (*n* = 2 < 5 years; *n* = 24 ≥ 5 years at follow-up). Two individuals reported receiving a diagnosis of ASD at < 1 year old which was unexpected given current diagnostic criteria, and therefore these individuals have been excluded from this table for ASD, but had additional co-morbid conditions and were therefore still included in the adverse neurodevelopmental outcomes group.

**Table 3:** Diagnosed neurological, neurodevelopmental, and sensory disorders

	Age at Follow-up		
	**< 5 years** (*n* = 48)	**≥ 5 years** (*n* = 145)	**Total** (*n* = 193)
**None**	**44 (92%)**	**72 (50%)**	**116 (60%)**
**1 + Diagnosed neurological**,** neurodevelopmental**,** or sensory disorder**	**4 (8%)**	**73 (50%)**	**77 (40%)**
Epilepsy	3 (6%)	27 (19%)	30 (16%)
Intellectual disability	0 (0%)	10 (7%)	10 (5%)
Cerebral palsy	0 (0%)	6 (4%)	6 (3%)
Vision impairment / Blindness	0 (0%)	6 (4%)	6 (3%)
Hearing impairment / Deafness	1 (2%)	3 (2%)	4 (2%)
Attention deficit/Hyperactivity disorder (ADHD)	0 (0%)	30 (21%)	30 (16%)
Learning disability (in reading, written expression, mathematics, or other specified impairment)	0 (0%)	27 (19%)	27 (14%)
Autism spectrum disorder (ASD)	1 (2%)	19 (13%)	20 (10%)
Language disorder / Auditory processing disorder	0 (0%)	7 (5%)	7 (4%)
Developmental coordination disorder	0 (0%)	3 (2%)	3 (2%)
Movement or tic disorder	0 (0%)	3 (2%)	3 (2%)

**Fig. 2:**
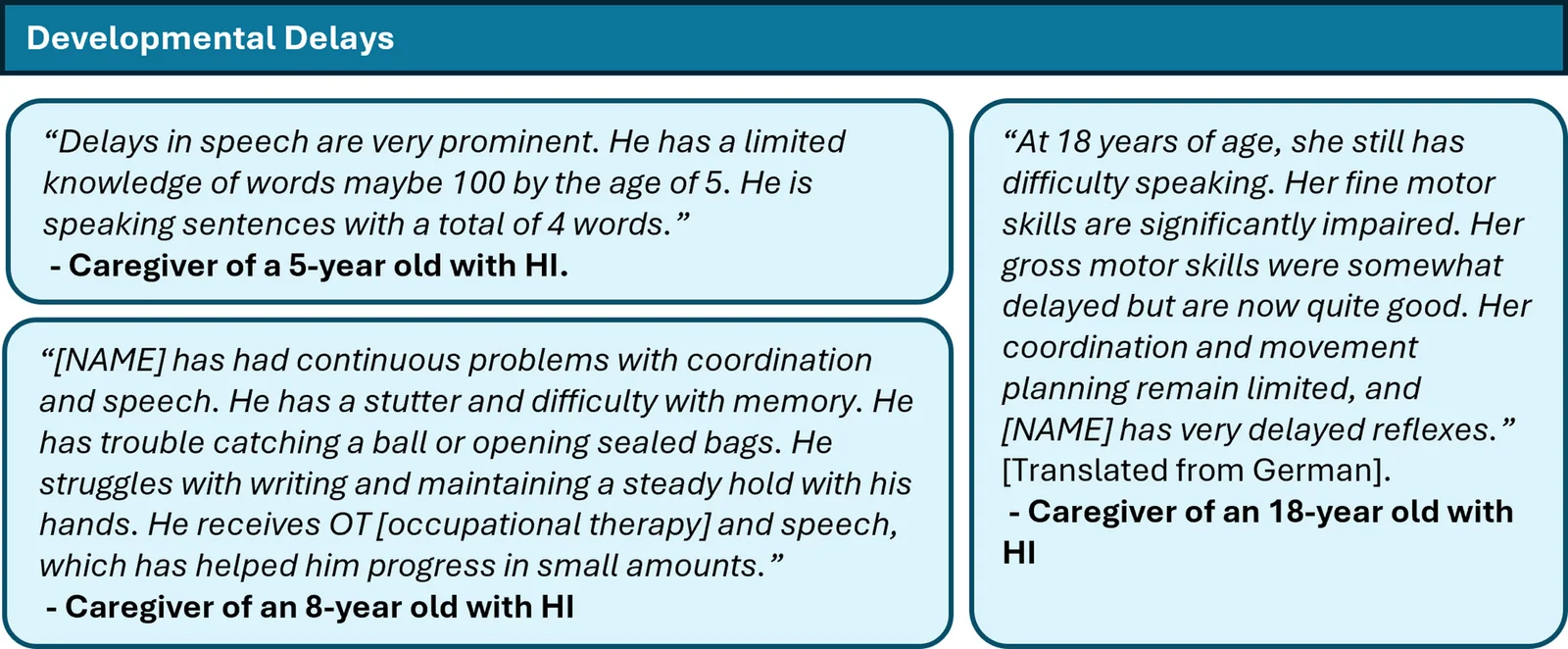
Free text comments on developmental delays

55% of individuals with HI reported experiencing delays in meeting developmental milestones (Table 4). Of the *n* = 105 individuals age ≥ 5 years with any adverse neurodevelopmental outcome, 10% reported developmental delays only (no diagnosed neurological, neurodevelopmental, or sensory disorders or other neurodevelopmental problems). Notably, 15% of respondents selected “other” developmental delay and the majority of these responses described delays related to feeding skills in the free text box (11%), however feeding issues are not typically considered to be a type of developmental delay and as such these were not included in the overall outcome. Many respondents used the free text box to elaborate further on the developmental delays experienced by the participant with HI (Fig. 2).

**Table 4:** Developmental delays (may not be formally diagnosed)

	Age at Follow-up		
	**< 5 years** (*n* = 48)	**≥ 5 years** (*n* = 145)	**Total** (*n* = 193)
**None**	**22 (46%)**	**58 (40%)**	**80 (41%)**
**1 + Developmental delay**	**25 (52%)**	**82 (57%)**	**107 (55%)**
Language/ speech/ communication milestones	7 (15%)	53 (37%)	60 (31%)
Gross motor milestones (e.g. sitting, crawling, walking)	17 (35%)	54 (37%)	71 (37%)
Fine motor milestones (e.g. grasping, pinching, ability to feed self)	6 (13%)	30 (21%)	36 (19%)
Cognitive milestones (learning, thinking, problem-solving)	2 (4%)	27 (19%)	29 (15%)
Social/ emotional milestones	2 (4%)	17 (12%)	19 (10%)
Coordination	2 (4%)	17 (12%)	19 (10%)
Other	11 (23%)	18 (12%)	29 (15%)
Unknown / Not reported	1 (2%)	5 (3%)	6 (3%)

In addition to a question about diagnosed medical conditions, respondents were also asked if the participant also experienced any other chronic problems. This question did not specify that the problem needed to be formally diagnosed. Of the list of problems provided, the most common issues reported amongst participants aged ≥ 5 years were learning disability (21%), poor coordination (21%), and sensory processing problems (21%) (Table 5).

**Table 5:** Other reported neurodevelopmental problems (may not be formally diagnosed)

	Age at Follow-up		
	**< 5 years** (*n* = 48)	**≥ 5 years** (*n* = 145)	**Total** (*n* = 193)
**None**	**33 (69%)**	**61 (42%)**	**94 (49%)**
**1 + Other reported problem**	**9 (19%)**	**68 (47%)**	**77 (40%)**
Learning disability	0 (0%)	31 (21%)	31 (16%)
Poor coordination	1 (2%)	31 (21%)	32 (17%)
Sensory processing problems	2 (4%)	30 (21%)	32 (17%)
Behavior problems	1 (2%)	23 (16%)	24 (12%)
Hypotonia	4 (8%)	16 (11%)	20 (10%)
Memory processing problems	0 (0%)	16 (11%)	16 (8%)
Movement disorder	0 (0%)	7 (5%)	7 (4%)
Unknown / Not reported	6 (13%)	16 (11%)	22 (11%)

**Fig. 3:**
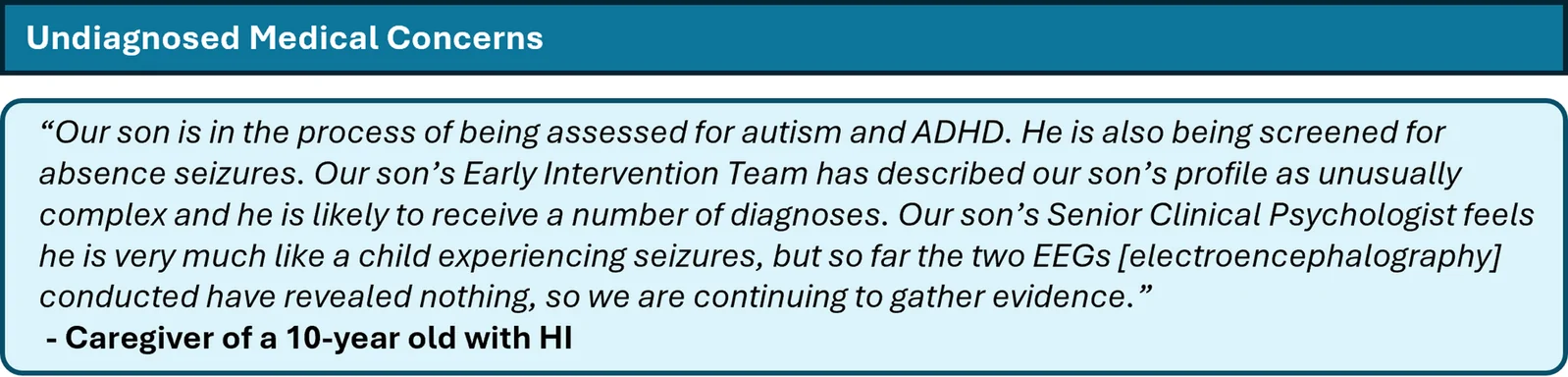
Free text comments on undiagnosed medical concerns

Respondents were also asked if they or the participant’s physician suspected the participant had any other conditions that were not yet diagnosed. 8% of participants with an adverse neurodevelopmental outcome and 15% of those aged ≥ 5 years with normal neurodevelopment at follow-up indicated suspicion of additional undiagnosed medical conditions, including both neurodevelopmental and non-neurodevelopmental conditions. Descriptions of undiagnosed problems described in free text included ADHD (*n* = 4, 25%), ASD (*n* = 2, 13%), seizures (*n* = 2, 13%), and learning disability (*n* = 1, 6%) (Fig. 3).

While not included in the definition of “adverse neurodevelopmental outcome”, it should be noted that 18% of participants aged ≥ 5 years reported a diagnosed mental health disorder, including anxiety disorder (16%), obsessive compulsive disorder (5%), and depressive disorder (3%) (Supplementary Table 1).

### Developmental evaluations and therapies

The majority of individuals who reported an adverse neurodevelopmental outcome reported that the participant had been evaluated by a developmental specialist (94%). Of those with normal neurodevelopment at follow-up, 38% of participants currently age ≥ 5 years and 65% of participants aged < 5 years reported that the participant had been evaluated by a development specialist. Half (49%) of participants with an adverse neurodevelopmental outcome reported seeing 5 or more development specialists. The most frequently reported specialists seen were speech therapist (63%), occupational therapist (57%), physical therapist (56%) and neurologist (53%).

Data was collected on whether participants experienced any access issues to developmental evaluations, however this question is newer and had a lower response rate. Of the 74 participants with an adverse neurodevelopmental outcome who completed this question, 39% reported experiencing some challenges with accessing developmental evaluations. Reported challenges included: not being offered an evaluation (12%), specialists not being available (9%), currently on a waitlist (9%), lack of insurance/healthcare system coverage (8%), and affordability (4%).

Beyond evaluation, 71% of participants with an adverse neurodevelopmental outcome reported receiving treatment for developmental delays or neurologic problems. Speech, occupational, and physical therapy were reported by 52%, 48%, and 43% of those with an adverse neurodevelopmental outcome, respectively.

### Comparison to peers

The *Development* survey also collected information on the participants’ ability to complete basic behaviors at an age-appropriate level and details surrounding accommodations for schooling. This analysis was completed for participants aged ≥ 5 years at follow-up only.

Many participants with an adverse neurodevelopmental outcome were described as performing below peer levels with regard to self-care, relationships, and cognitive tasks (Table 6). 17% of participants with an adverse neurodevelopmental outcome reported performing below peer level in all three domains and less than one-third (31%) of participants with an adverse neurodevelopmental outcome were performing at peer-level in all three domains.

**Table 6:** Comparison to peers and school accommodations

	Neurodevelopmental outcome (Age 5 + only)		
	**Adverse** (*n* = 105)	**Normal** (*n* = 40)	**Total** (*n* = 145)
**Capable of performing most ___________ as peers of the same age**			
**Self-care/Hygiene Behaviors**			
Yes	55 (52%)	38 (95%)	93 (64%)
Variable, depending on specific behavior	18 (17%)	1 (3%)	19 (13%)
No	29 (28%)	0 (0%)	29 (20%)
Unknown / Not reported	3 (3%)	1 (3%)	4 (3%)
**Relationship Behaviors**			
Yes	45 (43%)	38 (95%)	83 (57%)
Variable, depending on specific behavior	27 (26%)	1 (3%)	28 (19%)
No	31 (30%)	0 (0%)	31 (21%)
Unknown / Not reported	2 (2%)	1 (3%)	3 (2%)
**Cognitive Tasks**			
Yes	48 (46%)	39 (98%)	87 (60%)
Variable, depending on specific behavior	27 (26%)	0 (0%)	27 (19%)
No	27 (26%)	0 (0%)	27 (19%)
Unknown / Not reported	3 (3%)	1 (3%)	4 (3%)
**Performing at grade level or above in school**			
Yes	41 (39%)	29 (73%)	70 (48%)
Variable	23 (22%)	0 (0%)	23 (16%)
No	23 (22%)	0 (0%)	23 (16%)
Does not apply	1 (1%)	1 (3%)	2 (1%)
Unknown / Not reported	17 (16%)	10 (25%)	27 (19%)
**Attends a special needs school**	8 (8%)	0 (0%)	8 (6%)
**Home school or parent-taught**	4 (4%)	0 (0%)	4 (3%)
**Requires accommodations**,** modifications**,** assistive technology**,** or services at school**			
Yes	57 (54%)	9 (23%)	66 (46%)
No	32 (30%)	25 (63%)	57 (39%)
Unknown / Not reported	16 (15%)	6 (15%)	22 (15%)

**Fig. 4:**
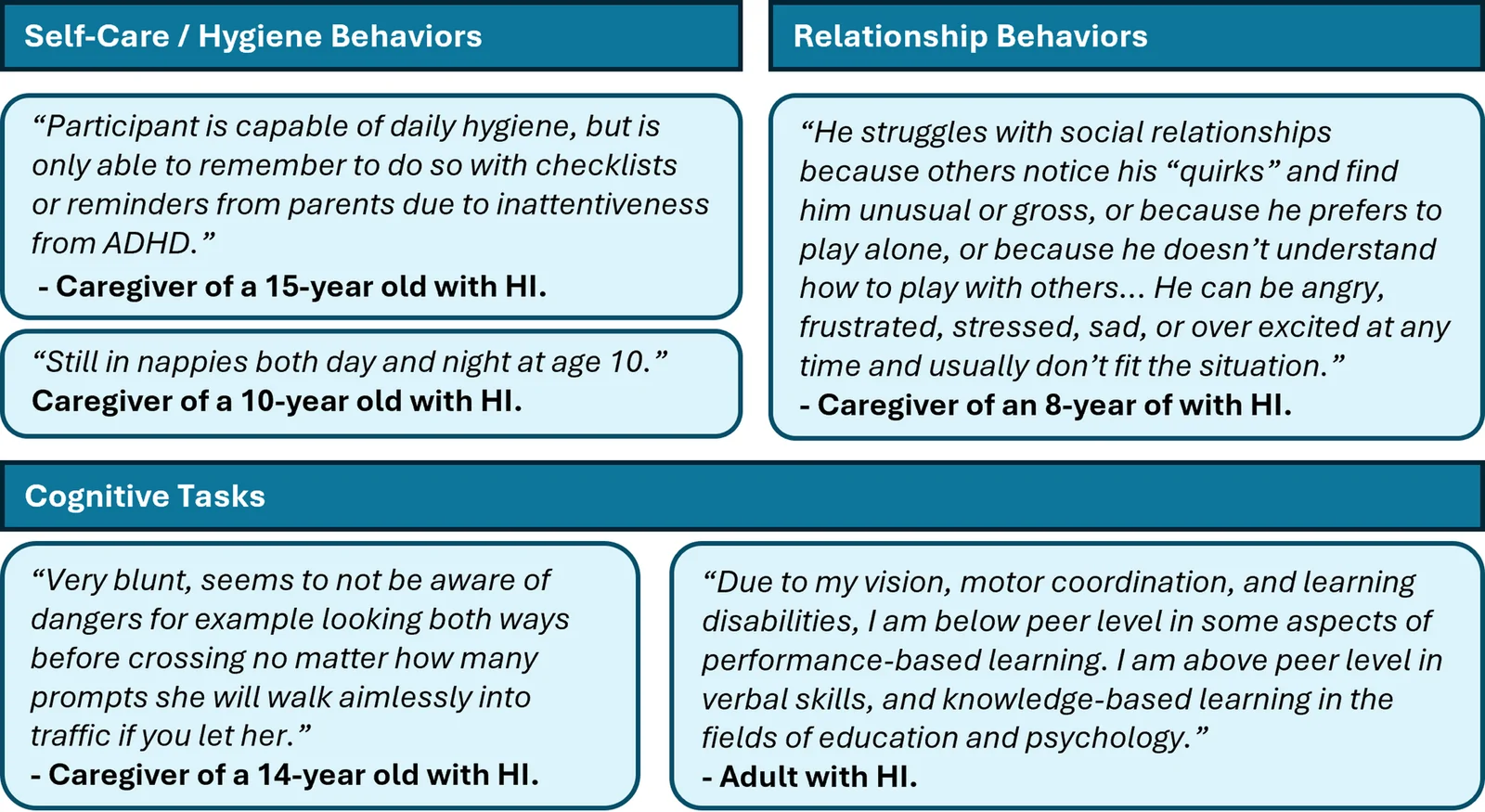
Free text comments on performing at peer level for self-care / hygiene behaviors, relationship behaviors, and cognitive tasks

Respondents were given the opportunity to elaborate on the participant’s ability to perform age-appropriate behaviors. Selected quotes focused on self-care and hygiene behaviors, relationship behaviors and challenges fitting in with peers, and cognitive tasks, including adult participants with HI who described areas where they excelled, or coping mechanisms used to overcome challenges (Fig. 4). Two parents (of participants age 8 years and 10 years) mentioned ongoing challenges with toilet training in free-text responses.

### School accommodations

**Fig. 5:**
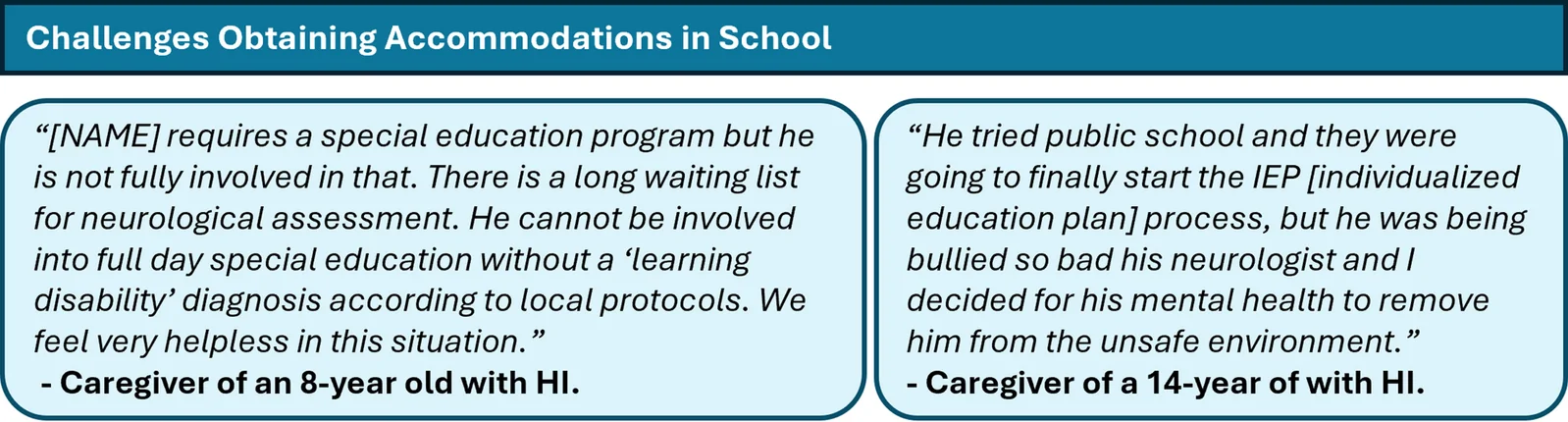
Free text comments on the challenges obtaining accommodations in school

Respondents were asked about the participant’s performance in school and any accommodations that were required. This analysis included all participants aged ≥ 5 years and included adult participants reflecting on their prior experiences at school. 39% of participants with an adverse neurodevelopmental outcome reported performing at grade level at school, 8% reported attending a special-needs school, and 4% were home-schooled or parent-taught (Table 6). None of the participants with normal neurodevelopment reported performing below grade level or attending a non-mainstream school. 54% of participants with an adverse neurodevelopmental outcome reported receiving accommodations, modifications, assistive technology, or services at school. When asked to describe the specific support that was received, respondents described a variety of individualized accommodations that were specific to each participant’s needs. These included having a formal individualized education plan, extra time for tests and completing school work, having a classroom aide or teaching assistant, working in small groups or 1:1 support, larger print, additional help with writing and spelling, assistive technology including audio books, computer access, speech-to-text and predictive text tools, and communication devices. Overall, 61% described educational accommodations in the free text, 25% described requiring a teaching assistant or personal aide (some of which may be for medical support rather than educational support), and 16% described requiring social accommodations. Some respondents elaborated further on the challenges associated with obtaining the required accommodations (Fig. 5).

## Discussion

This is the first study of neurodevelopmental outcomes in people with HI to focus on real-world data as reported directly from people with HI and their families. With over 190 participants, this study also represents one of the largest cohorts reporting on neurodevelopmental outcomes in HI. This is an important addition to the current literature, as previous reports focused on clinical assessments may not fully capture the breadth of challenges experienced by people with HI and may underestimate the true prevalence of neurodevelopmental challenges in this population. This study brings the patient and caregiver voice directly to the growing body of data on neurodevelopmental challenges in HI, and the free text quotes add richness to the depth of understanding on daily challenges.

This study found a prevalence of adverse neurodevelopmental outcomes of 66% of all study participants, or 72% of those age ≥ 5 years old, which is higher than prior prevalence estimates of ~ 20–50% [2, 16, 17, 19, 21–23, 25]. This higher estimate reflects the more inclusive nature of this study to acknowledge a broader range of neurodevelopmental challenges experienced by people with HI, which may be underestimated in prior studies focused on clinical assessments alone. This may also reflect the selection bias of registry participation, whereby individuals with more severe disease may be more inclined to participate in research than those with milder cases.

Around one in eight participants reported normal neurodevelopment but were under 5 years old at the time of follow-up, which may be too young to determine if there is an adverse neurodevelopmental outcome. This is because many neurodevelopmental differences may not be evident or diagnosed until later childhood.

Mannisto et al. [17] suggested that long-term neurological outcomes have improved in the 21st century, based on a Finnish population-wide cohort study from 1972 to 2015 showing that the percentage of individuals with normal neurodevelopmental outcomes was significantly greater for those diagnosed with HI after the year 2000 compared to those diagnosed earlier. However this has not been demonstrated in other studies [24] and prevalence estimates from more recent studies mirror those from older studies [2, 19, 21–23].

Expert discussion (unpublished) amongst the members of the Congenital Hyperinsulinism International Collaborative Research Network (CHI CRN) [34] suggests that the incidence of severe adverse neurodevelopmental outcomes such as cerebral palsy, epilepsy, vision impairment, hearing impairment, and intellectual disability has declined in recent years, while the incidence of milder neurodevelopmental challenges such as behavioral issues has increased. This hypothesis has not been formally studied at this time but may explain the higher overall rates of adverse neurodevelopmental outcomes in this study compared to others. In this study, around 1 in 6 individuals reported epilepsy, but fewer (2–5%) reported cerebral palsy, vision impairment, hearing impairment, and intellectual disability; the majority of which were ≥ 5 years old at follow-up. This is similar to the published literature on the prevalence of these outcomes: epilepsy (9–31%), cerebral palsy (2–13%), vision impairment (3–5%), hearing impairment (3%), and intellectual disability (4–7%) [2, 16–19, 21, 23]. It is difficult to assess the prevalence of “milder” neurodevelopmental challenges in this study, as the concept of severity is largely subjective and was not captured within the registry surveys. However, a higher proportion of individuals with HI in this study did report autism (10%) and ADHD (16%) compared to the general US population of children aged 3–17 years (3% and 11%, respectively) [35, 36].

A common, underreported aspect of HI is the prevalence of mental health conditions within the HI community. In this study, 16% of individuals with HI age ≥ 5 years reported being diagnosed with an anxiety disorder; compared to 7% in the general US population of children aged 3–17 years [37]. While this may not be a direct neurological consequence of hypoglycemia, it may indicate the significant impact of fear of hypoglycemia and HI-related medical trauma on people with HI, even from a younger age.

Developmental delays were highly prevalent in this study, reported by over half of participants. This is comparable to the 51% of caregivers who reported concerns about the participant’s development in the study by Sigal et al., [2], but higher than other studies that focus more on clinical assessments than patient or caregiver perspectives (17–32%) [16, 19]. This is also much higher compared to the general US population of children aged 3–17 years [35]. Of note, many caregivers in this study reported “feeding issues” in the free text box for “other” developmental delays. This is not typically considered to be a developmental delay and as such was not provided as a predefined response option within the survey and was not included in the definition for the overall adverse neurodevelopmental outcome, however many families felt it was impactful enough to be mentioned. Although the authors do not consider feeding issues to be a neurodevelopmental issue, feeding issues are known to be a prevalent issue amongst people with HI, affecting around 70% of HIGR participants [11, 29]. It is challenging to determine whether these issues reflect an underlying neurological problem or are related to the treatments required to manage glycemia (i.e. tube feeding, medications, etc.) and this is an understudied aspect of life with HI that still requires further research.

Half of participants with an adverse neurodevelopmental outcome reported receiving evaluations from ≥ 5 different developmental specialties, such as speech therapist, occupational therapist, physical therapist, neurologist, etc., indicating the breadth of challenges experienced by people with HI and the importance of obtaining well-coordinated multi-disciplinary care across specialties. The International Guidelines for the diagnosis and management of HI recommend that all children with HI should receive regular neurodevelopmental follow-up and monitoring, including formal testing [12]. There is limited data on the prevalence of access issues to developmental evaluations for people with HI, but data from this study indicates that while a great number of participants can access developmental evaluations, many families experience issues accessing the care they need. There is a need for proactive referrals to developmental specialists for all children with HI, in addition to ongoing evaluations as the child grows to ensure they are developing appropriately in all stages of childhood and adolescence. In some US states and other countries around the world, babies born prematurely may qualify for early intervention programs to monitor their developmental progress prior to the development of any known delays [38, 39], however this is not currently standard practice for all babies who experience hypoglycemia. Of participants with normal neurodevelopment, more participants who were < 5 years at follow-up reported developmental evaluation compared with those age ≥ 5 years (65% vs. 38%). This may already indicate shifting practices towards evaluating children with HI for developmental challenges in the absence of known problems, however a gap in assessing all children remains.

Almost all participants aged ≥ 5 years with a normal neurodevelopmental outcome reported performing at or above peer level in self-care/hygiene behaviors, relationship behaviors, and cognitive tasks, compared to half or less of participants with an adverse neurodevelopmental outcome. This data is especially unique within the HI literature; no other study reports on subjective assessments of how well the person with HI is able to function in these basic domains of daily life compared with their peers. This data highlights the gap between those with and without adverse neurodevelopmental outcomes and the real-world impact of HI in a way that formal clinical testing has not. It is important to note that while many individuals are struggling, there are also many individuals with an adverse neurodevelopmental outcome who reported performing at grade level in school and at peer level or above for various daily tasks. Further study is required to assess whether this is due to milder neurodevelopmental challenges, or due to early identification of challenges, therapy, and support. The ability to function at peer level is an important area for people with HI to receive support in, as this will significantly impact their mental health and overall quality of life.

When asked to describe the challenges the participant experienced, respondents described a range of physical and social challenges. Of note, two respondents with older children (8–10 years old) mentioned ongoing issues with toilet training. As this was not a defined question within the survey, we cannot accurately quantify the extent of problems with toilet training in this population. However, this warrants further study to determine if this is a developmental challenge, a physical/neurological problem, or a consequence of increased fluid intake necessary to manage HI (e.g. continuous dextrose).

Half of individuals with an adverse neurodevelopmental outcome reported receiving accommodations, modifications, assistive technology, or services at school, of which one-quarter described requiring an aide or assistant at school. Of those who did not say that they received any type of accommodation at school, it is unclear if this is because they did not feel they needed accommodations, or if it was due to problems accessing and advocating for such accommodations. As such, this data may reflect an underestimate of the true need for additional support services at school. This represents a significant burden of disease and cost of social support services for people with HI, which could be mitigated by earlier diagnosis and intervention to limit or prevent hypoglycemia and subsequent brain damage.

### Strengths & limitations

This is a registry-based study presenting patient- and caregiver-reported insights on neurodevelopmental outcomes in people with HI, and as such this study is limited by factors that are inherent to registry-based research, particularly in a rare disease. Study recruitment was conducted primarily via the advocacy organization, Congenital Hyperinsulinism International, and so recruitment was biased towards those who were more connected to the HI community. This is also reflected in the underrepresentation of participants with cured focal HI or otherwise resolved HI in HIGR, likely due to a lower level of research engagement for individuals who are considered cured or resolved. HIGR is an online registry, which selects for participants who have access to technology and internet to complete the surveys. Respondents could choose to complete surveys at their own pace and in any order, and so registry participants who had not completed the surveys of interest were excluded from this study, which may have biased the study population to those who were more willing to complete these surveys. The data collected via registry surveys is limited to what the respondent chooses to share and may be missing data that they are not comfortable sharing. The registry does not collect data on the severity of each outcome and therefore we could not objectively categorize some individuals as having more severe outcomes than others. The surveys collect data retrospectively, and so data may be impacted by recall bias, especially from older participants who may be recalling events from many years prior. Efforts have been made to improve the longitudinal data collection capabilities of HIGR, but it is possible that not all questions within a survey were updated by the respondent, leading to outdated data at follow-up. However, this would lead to an underestimate of the extent of neurodevelopmental challenges. The insights shared through free-text responses are limited to what the respondents choose to share, with some participants sharing a lot of detail and others choosing not to write anything. It is possible that other participants experienced the same issue or challenge but did not share this, leading to an underestimate of the frequency of themes that were quantified from the free-text responses. Finally, this study is limited by the absence of a control group. However, as the study design was real-world and observational, a concomitant control group was not deemed suitable.

Despite these limitations, this study has a number of strengths that set it apart from other studies in the current literature. The online nature of the registry can also be considered a strength, as this reduces the barriers to participation around the world compared to in-person studies that are limited by geography and time commitments. HIGR is available in eight languages, improving global participation. As such, this study represents the most diverse study on neurodevelopmental outcomes in HI: with a large international cohort of participants treated at different types of medical centers in 34 countries around the world, the results of this study are more generalizable than many other studies. Most importantly, self- and parent-reported data on the presence of a wide range of neurodevelopmental, social, and behavioral problems allowed the capture of all challenges in daily life. This data provided a much richer understanding of challenges beyond what can be diagnosed through formal clinical assessments. The inclusion of free-text responses is a particular strength of this study, as these insights provide detail and nuance to the categorical data, allowing much deeper understanding of the challenges experienced by families. Furthermore, these responses were driven by what each individual or family felt was most important to them, rather than which assessments clinicians chose to administer.

## Conclusions

This is the first study of neurodevelopmental outcomes in people with HI to focus on real-world data as reported directly from people with HI and their families. Neurodevelopmental challenges were highly prevalent in people with HI and extended beyond formal clinical diagnoses into social, emotional, and behavioral problems. This study found high rates of autism and ADHD, and is the first to report on mental health disorders in people with HI. The impact of timing and severity of hypoglycemia on subsequent neurodevelopmental outcomes in HI is still unknown and requires further study, but faster diagnosis and appropriate early treatment may help to limit or prevent neurodevelopmental challenges. Proactive developmental assessments should be offered to all children with HI, and efforts should be made to develop multidisciplinary teams to support all medical and developmental aspects of care for people with HI. The real-world data on neurodevelopmental outcomes and experiences of individuals with HI and their families generated in this study will serve as a benchmark for future studies and will help to quantify how newer advances in diagnosis and treatment impact patient-centered neurodevelopmental outcomes.

## Supplementary Information

Below is the link to the electronic supplementary material.

Supplementary Material 1

## Data Availability

The datasets generated and/or analyzed during the current study are not publicly available because HIGR is an open and active registry, with new data being added regularly. Reasonable requests for updated data from the HI Global Registry can be made to info@higlobalregistry.org. Declarations.

## Footnotes

1 The association of UCP2 with HI is a debated topic, with some reports describing its association with HI [31, 32] and another stating that it does not cause HI [33].

## Abbreviations

ADHD: Attention deficit hyperactivity disorder
ASD: Autism spectrum disorder
CHI: Congenital Hyperinsulinism International
HI: Congenital hyperinsulinism
HIGR: HI Global Registry
